# Real-Time Avalanche Hazard Monitoring System Based on Weather Sensors and a Laser Rangefinder

**DOI:** 10.3390/s25092937

**Published:** 2025-05-07

**Authors:** Natalya Denissova, Olga Petrova, Erbolat Mashayev, Dmitry Spivak, Vitaly Zuyev, Gulzhan Daumova

**Affiliations:** 1 Department of Information Technology, D. Serikbayev East Kazakhstan Technical University, Ust-Kamenogorsk 070000, Kazakhstan; ndenisova@edu.ektu.kz (N.D.); ymashayev@ektu.kz (E.M.); vzuev@ektu.kz (V.Z.); 2 School of Geosciences, D. Serikbayev East Kazakhstan Technical University, Ust-Kamenogorsk 070000, Kazakhstan; gdaumova@edu.ektu.kz

**Keywords:** monitoring system, software–hardware complex, measuring equipment, microcontrollers, avalanche hazard

## Abstract

Avalanche hazard prediction remains a crucial task for mountainous regions worldwide. This study presents the development and field testing of a prototype automated avalanche hazard monitoring system designed for the East Kazakhstan region. The system integrates a snow avalanche station (including temperature, humidity, and pressure sensors; a magnetoelectric wind sensor; a data logger; and devices for autonomous operation), a temperature snow measuring rod, an API (application programming interface) service for storing weather and climate parameters in a database, and a web interface. Powered by autonomous solar energy solutions, the system ensures continuous, high-resolution monitoring of key environmental parameters. Using initial test datasets, we analyzed the specific strengths and weaknesses of the developed monitoring system using the example of one avalanche site. Avalanche prediction was performed using regression analysis (logistic regression). The evaluation of the model showed a high forecasting accuracy, with recognition rates exceeding 98%. The obtained regression coefficients can be used to predict avalanches based on meteorological data collected using the proposed equipment. The developed solution holds significant promise for improving avalanche risk management practices and can be expanded for broader application in both national and international contexts.

## 1. Introduction

Since the task of predicting avalanche hazards is relevant and in great demand for many countries, today these studies and the development of automated systems are carried out almost all over the world. Various equipment can be used to create such systems, from acoustic devices to space satellites. Methods and approaches to solving the problem are also diverse.

Among the developed monitoring systems, a comprehensive web-based avalanche hazard monitoring system created by researchers from France [1] can be noted. The authors recommend using three data sets for forecasting: the avalanche chronicle (Enquête Permanente sur les Avalanches, EPA), avalanche maps (Carte de Localization des Phénomènes d’avalanche, CLPA) and a collection of data on hazards for populated areas. The data are integrated into a common database, which ensures full compatibility between all types of avalanche records: digitized geographical data, avalanche characteristics, eyewitness reports, photographs, hazard and risk levels.

Avalanche analysis based on remote sensing data and detailed field surveys is proposed by Chinese authors Bian et al. The researchers studied the mountainous regions of Sichuan Province, China [2]. A similar avalanche analysis based on remote sensing data is used by various researchers. These methods give good results in the identification of avalanches based on remote sensing [3].

Many researchers use remote sensing data from the Sentinel-1 satellite. Thus, the authors Yang et al. [4] performed an avalanche hazard assessment based on the analysis of images obtained from the Sentinel-1 satellite as part of their study. To predict avalanches in the mountains of Western Tien Shan (China), the authors have developed a mathematical model including six indices. In the work of other authors [5], an automatic avalanche monitoring system operating in real time is presented. This system detects avalanche hazards by comparing Sentinel-1 image data for five years in Northern Norway. In [6], methods for remote avalanche detection are described with the refinement of remote sensing data due to the long interval of their reception from Sentinel-1 satellites. This study suggests a new approach to automating avalanche detection by analyzing video streams from surveillance cameras using a machine learning module.

The authors of Edme et al. propose using distributed acoustic sensing data based on fiber-optic telecommunication cables to study the possibility of avalanches [7]. Another method of predicting an avalanche is based on the use of infrasound waves; it is provided by researchers Marchetti et al. in [8]. The proposed methodology is based on the assessment of threshold criteria for sources of infrasound waves and the determination of possible avalanches based on them.

When conducting avalanche research in a particular area, it is necessary to identify possible avalanche sites, calculate their parameters and determine the avalanche regime. For this purpose, observations of avalanches and indirect signs of avalanche danger are used, statistical dependencies and mathematical models are built, and archival data are studied. Avalanche hazard maps are compiled based on the collected data.

They are the main tool for avalanche safety management in mountainous areas [9]. Avalanche hazard indication mapping has previously been tested in various regions of the world. In Switzerland and most other Alpine countries, avalanche hazard maps are compiled by avalanche experts for individual avalanche routes based on historical data, field research, terrain analysis, forest information, and numerical modeling.

Turkish researchers have built avalanche hazard maps [10,11] for several regions in the country based on Digital Terrain Models. Other researchers [12] have created an avalanche hazard map using Geoinformation System (GIS) tools and collecting information from newspapers, field observations, and user reviews from remote areas. The widespread use of GIS and remote sensing of the earth for solving applied problems makes it possible to create predictive models and maps [13,14,15].

The work of other researchers [16,17] reflects the development of new methods for mapping and indicating avalanche hazards, taking into account the topography of areas and using machine learning methods.

The authors of [18] proposed an automated approach to creating avalanche hazard indication maps for large regions based on Digital Terrain Model data. They calculated eight different scenarios with periods of recurrence from frequent to very rare avalanche events, with and without forest protection. The proposed approach combines the automatic detection of potential avalanche-prone areas and their dynamics in numerical simulation. The resulting spatially consistent avalanche hazard indication maps were then checked for compliance with existing official risk maps based on satellite data sets.

The authors of [19] have shown that, in general, mapping avalanche hazards for specific territories and the magnitude of events is not an easy task. Special mention should be made of the authors [20] who developed an avalanche warning system and a real-time navigation system based on a global positioning system (GPS) in a GIS environment. This system is used to visualize terrain maps and navigate in avalanche-prone regions of India. The maps show the location of camps, avalanche-prone areas, hiking routes, as well as information about the climatic conditions of the area. The development was integrated into an online platform for visualizing geospatial data, periodically updating snow conditions, and predicting avalanche sites. They also developed an avalanche warning and real-time navigation system based on GPS [21].

The authors Xi N., Me G. [22] in their work clarified the spatial and temporal characteristics of snow cover and mapped them on maps, as well as topography, meteorology and vegetation data. They used satellite images for this purpose.

Later, other researchers [23] developed statistical models for local avalanche forecasting. Numerical one-dimensional models such as AVAL-1D, Voellmy-Salm, or VAlanghe RAdenti (VARA) [24] are widely used in Switzerland and other countries in Europe, America, and Asia. Their limitations in open and difficult terrain have led to the development of 2D and 3D models such as SAMOS, developed for modeling dry avalanches [25] or RAMMS (Rapid Mass Movement Modeling) [26].

However, some authors [27] argue that studies of avalanches using RAMMS without taking into account accurate data on the height of the snow cover, the general properties of snow and local meteorological conditions do not provide a complete picture of avalanche danger. They studied the avalanche situation using the example of the western Himalayas. In parallel with the development of various models, numerous studies have explored the possibilities of remote sensing for the detection and investigation of avalanches [28]. Remote sensing is widely used to map avalanches in various ways [29]. For example, this can be performed by manually digitizing avalanches with high resolution from the initiation zone to the descent zone [30]. It is known that both optical remote sensing [31] and synthetic aperture radar technology [32] are used in avalanche forecasting. The method of automatic detection from radar images is also known [33]. On the Tien Shan, researchers [34] used interpretation of remote sensing images of the Earth and drone photography in conjunction with field research to determine avalanche hazards.

There are remote avalanche monitoring systems that can use various equipment. Thus, researchers [2] compared three such systems: an avalanche radar, infrasound array system consisting of four infrasound sensors, and single sensor infrasound system.

The authors of [35] investigate a computer vision-based notification system. Other researchers use acoustic devices and video surveillance as part of software and hardware complexes to detect and monitor avalanches. They allow real-time detection of avalanches that can block traffic on highways. Tests have confirmed the effectiveness of such complexes. The level of false alarms, according to one study, was estimated to be within 13–30% [36,37,38,39].

The review (Table 1) showed that various methods and approaches can be used to monitor avalanches. To obtain the highest-quality forecast, a combination of several methods, the use of ground-based observations and remote sensing, and data mapping can be used.

Thus, the studies presented above mainly rely on global data sources (such as Sentinel-1, Sentinel-2 imagery, RAMMS modeling, and others), while there are very few studies focused on local automated systems for data collection and processing in avalanche-prone areas. In most cases, these are automated systems that collect only meteorological data, with no direct measurements of snow cover thickness.

Nevertheless, the reviewed studies indicate a rising interest in autonomous avalanche monitoring systems. The growing need for detailed and timely data collection emphasizes the importance of continued development and implementation of such technologies.

The purpose of this study is to develop a module for an automated avalanche hazard system with sensors on slopes. We offer a ground-based measurement system that will allow for permanent monitoring of the distribution of meteorological parameters and snow depth in avalanche zones for the East Kazakhstan region (Kazakhstan) with high spatial and temporal resolution. This complex will be the core of the avalanche monitoring and forecasting system.

## 2. Materials and Methods

In this study, we completed the development of a software and hardware complex and tested it under real conditions at one of the avalanche-prone sites in Eastern Kazakhstan. A base meteorological station equipped with sensors for measuring wind speed and direction, air temperature, humidity, atmospheric pressure, snow cover depth, and the temperature gradient within the snowpack was deployed. In addition, solar panels, rechargeable batteries, charge controllers, and LTE/Wi-Fi routers were used to ensure autonomous operation and reliable data transmission.

We proposed a complex consisting of autonomous hardware located on the slope (in the avalanche collection area) and an analytical system for processing the collected data. Logistic regression was used to analyze the data and make forecasts. The primary method involved establishing permanent ground-based monitoring of meteorological parameters and snowpack conditions using an automated data collection system. Sensor data were transmitted in real time via LoRa and LTE/Wi-Fi networks to the AvaAPI server for subsequent analysis and avalanche hazard forecasting.

As part of the study, a prototype of an autonomous automated avalanche hazard monitoring system with the capability for early prediction and prevention of avalanches was created.

## 3. Results and Discussion

**Figure 1:**
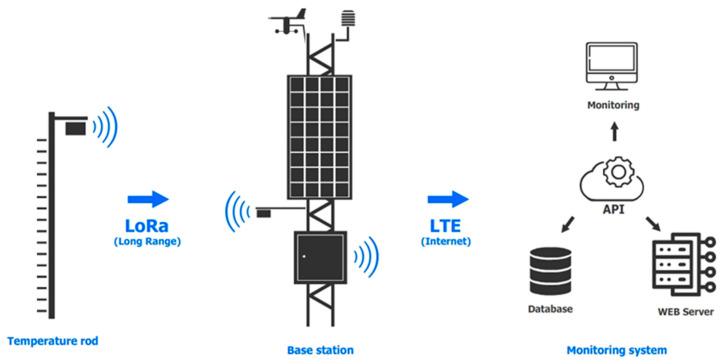
Avalanche hazard monitoring system diagram.

The main components of the system are (Figure 1) avalanche station; temperature snow measuring rod; API (application programming interface) service for saving weather and climate parameters in the database; web interface of the avalanche danger monitoring system.

The avalanche base station is a software and hardware complex consisting of sensors and equipment that collect information on key weather and climate parameters to ensure effective monitoring and data transmission in real time. The list of equipment installed on the prototype of the avalanche station is presented in Table 2. The list of received weather and climate parameters includes wind speed; wind direction; air temperature; relative air humidity; atmospheric pressure; snow cover height; temperature gradient of the snow cover.

The system has one limitation—data transmission problems that occur in case of interruption of transmission over cellular communication channels that depend on the service provider. This problem can be solved by using two or more telecom operators at the same time.

**Figure 2:**
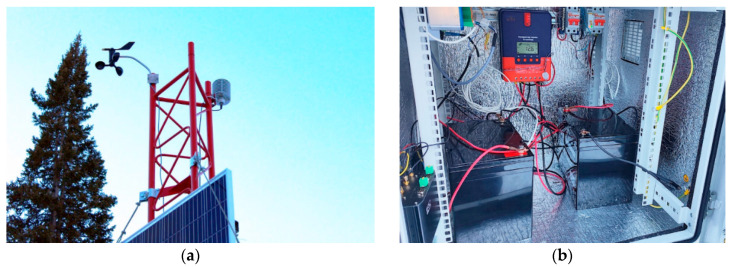
Placement of sensors and probes at the base station: (**a**) wind sensor and temperature, humidity and pressure sensor; (**b**) outdoor all-weather cabinet equipment.

The source of wind speed and direction data is the DBM-6410 Wind Sensor. The Temperature–Humidity–Pressure Sensor (Lambrecht THP[pro]) (Figure 2a) in the Sensor shelter is used to measure temperature, humidity and atmospheric pressure. The rest of the equipment is housed in a weatherproof outdoor cabinet (Figure 2b).

Sensor readings are transmitted to the data logger (Lambrecht Ser[LOG] Plus) via the Modbus protocol. This logger supports connection and operation with a variety of different sensors—from temperature and humidity to more complex meteorological sensors such as barometers, anemometers and other devices. The device supports various connection methods, including RS-232 (Recommended Standard 232—physical layer standard for asynchronous interface), RS-485 (Recommended Standard 485—physical layer standard for asynchronous interface), USB (Universal Serial Bus—industry standard for digital data transmission and power delivery between many types of electronics) and analog inputs. The logger can collect data in real time, as well as support periodic or event-based data collection modes. It can store data in its memory or transmit it to an external server. Below is the code executed on an LTE (Long-term evolution is a standard for wireless broadband communication for cellular mobile devices and data terminals)/Wi-Fi (A way of connecting to a computer network using radio waves instead of wires) router, implementing the functionality of a proxy server that accepts HTTP (Hypertext Transfer Protocol is an application layer protocol in the Internet protocol suite model for distributed, collaborative, hypermedia information systems) requests on the local host, changes the request header parameters and forwards it to a remote API (Application Programming Interface is a connection between computers or between computer programs) server using an SSL (Secure Socket Layer is a security protocol that lets applications encrypt information that moves between a client to a matching server) connection. Below is the code running on an LTE/Wi-Fi router that implements the functionality of a proxy server that accepts HTTP requests on the local host, modifies the request header parameters, and forwards it to a remote API server using an SSL connection (Table 3).

All sensor readings aggregated by the recorder are converted into a JSON (JavaScript Object Notation is a text-based data interchange format based on JavaScript) format convenient for transmission, and are sent every 15 min via HTTP to the AvaAPI (API service, developed within the framework of the current project). The AvaAPI server accepts the request, performs authorization, validates the transmitted data and saves them in the database for further analysis (Figure 1). The process of data transfer between the logger and the API service is provided via an LTE Internet connection through an iRZ RL25w router. The autonomy of the system is ensured by the use of an OSDA Solar 380M ODA380-30-MH (OSDA Solar established, Ningbo, China) solar panel with a peak power of 380 W, operating in conjunction with two batteries with a capacity of 75 Ah each. Battery charging is controlled via the SRNE SR-ML2420 MPPT controller (SRNE Solar, Shenzhen, China), which optimizes the performance of the solar panel. The main equipment, with the exception of the sensors, is housed in an all-weather 12U outdoor cabinet (Figure 2b), which protects the equipment from adverse weather conditions and maintains the specified temperature regime.

**Figure 3:**
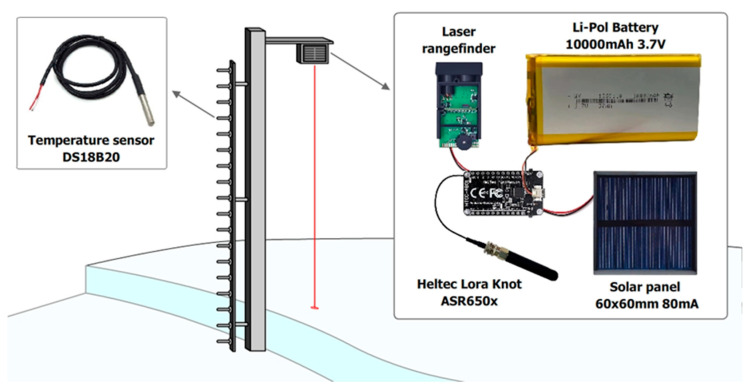
Temperature snow gauge.

The cabinet itself with the equipment and sensors is mounted on a truss mast structure six meters high. In addition to weather and climate parameters, information on snow cover is important for monitoring and forecasting avalanche danger. The collection of these parameters is provided by snow temperature rods (Figure 3), and a Heltec LoRa 32 microcontroller (Heltec Automation, Chengdu, China) paired with a laser rangefinder installed directly on the base station, which is a receiver of data from snow measuring rods.

Temperature snow measuring rods are autonomous devices integrated into the monitoring system. Each rod is based on the CubeCell—AB01 Dev-Board (V2) (Heltec Automation, Chengdu, China) microcontroller and is equipped with a number of key components (Figure 3) for collecting and transmitting data. The software code of the microcontroller on the temperature snow measuring rod collects data from the laser rangefinder and a number of temperature sensors, and sends them via LoRaWAN (Heltec Automation, Chengdu, China) (long-wave radio communication) to the base station receiver with a frequency of 15 min (Table 4).

Below is the code executed on the temperature rod data receiver (Table 5). When data are received from the rail, the radio module reads the incoming data. Then, a packet string is formed, which includes information about the received data and the measured distance obtained from the laser rangefinder installed on the receiver. Next, a data packet is formed, encoded in base64 and sent to the target server over the Wi-Fi network via an HTTP POST request.

**Figure 4:**
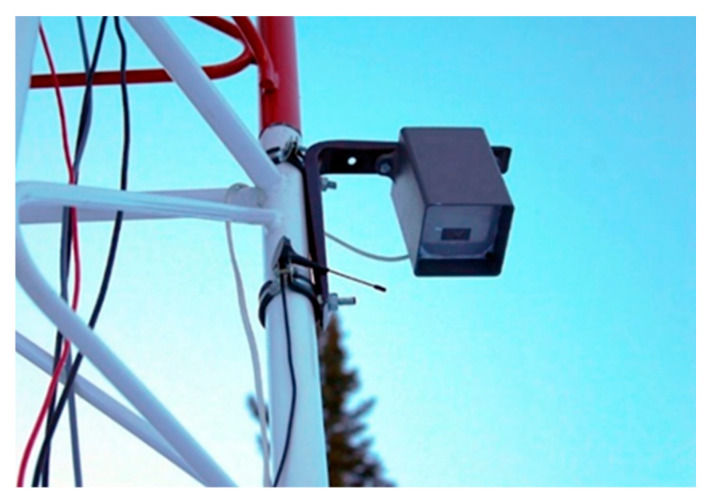
Receiver of data from a temperature snow measuring rod.

The temperature snow measuring rod includes: a HI50 laser rangefinder designed for high-precision measurement of snow cover depth; 18 DS18B20 temperature sensors installed on a vertical rod with a 10 cm step, which allows obtaining detailed data on the temperature layers of the snow cover; two lithium-polymer batteries with a capacity of 10,000 Mah, providing energy autonomy for the device; two 60 × 60 mm solar panels with an output current of 80 mA, which maintain the battery charge and extends the life of the device in autonomous mode. Temperature snow measuring rods collect data on snow depth, snow cover temperature gradient and battery voltage. The collected data are transmitted every 15 min using the LoRa (Long Range) wireless communication protocol at a frequency of 433 MHz to the receiver (Figure 4) located at the base avalanche station. The role of the receiver at the base station is performed by the Heltec LoRa 32 microcontroller, which is also equipped with an HI50 laser rangefinder. This allows the device to combine the functions of measuring the height of the snow cover and receiving data from temperature rails. The microcontroller aggregates the data in its memory and transmits them to the AvaAPI API service at 15 min intervals. Data are transmitted via a Wi-Fi connection between Heltec LoRa 32 and the iRZ RL25w LTE/Wi-Fi router (iRZ Electronics Company, Saint Petersburg, Russia), which provides an Internet connection.

In general, the prototype of the avalanche hazard monitoring system consists of a base station—a mast with mounted equipment and a temperature snow measuring rod. In this case, several snow measuring rods can be installed in an avalanche-prone area. The base station can be installed within the boundaries of an avalanche-prone area near the avalanche catchment area. This will ensure the collection of relevant meteorological data for the area and the safety of the base station from an avalanche. Temperature snow measuring rods, on the contrary, can be installed in places where snow height control is necessary, for example, directly in the avalanche catchment area or in the zone of active wind snow transfer.

**Figure 5:**
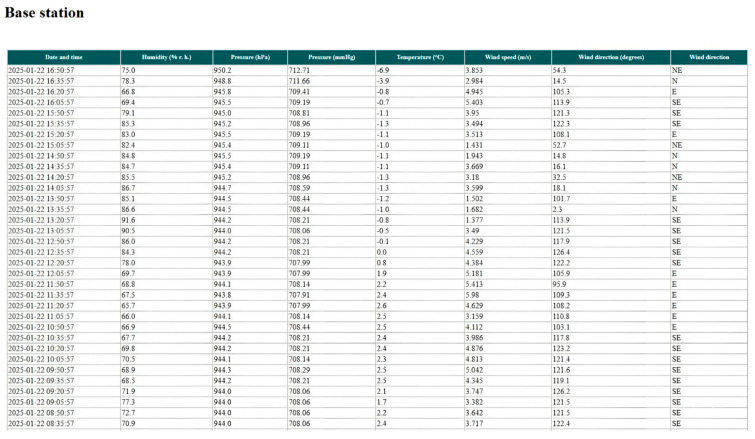
Example of base station data.

As a result of the operation of the base station and the snow measuring rod, the following data are transmitted to the database every 15 min from meteorological sensors installed at the base station: humidity (% rh); pressure (hPa); pressure (mmHg); temperature (°C); wind speed (m/s); wind direction (Figure 5).

**Figure 6:**
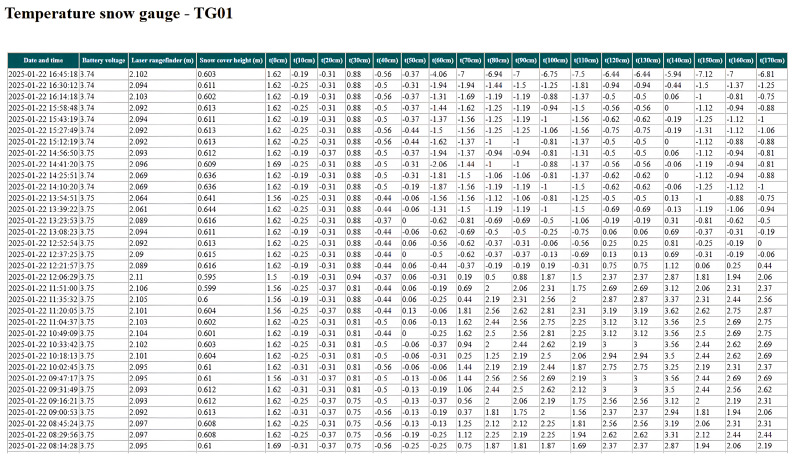
Example of snow measuring rod.

Data from the sensors of the snow measuring rod are also transmitted: battery voltage; laser rangefinder (m); snow cover height (m); temperature from 18 sensors installed on a vertical rod with a 10 cm step (Figure 6).

**Figure 7:**
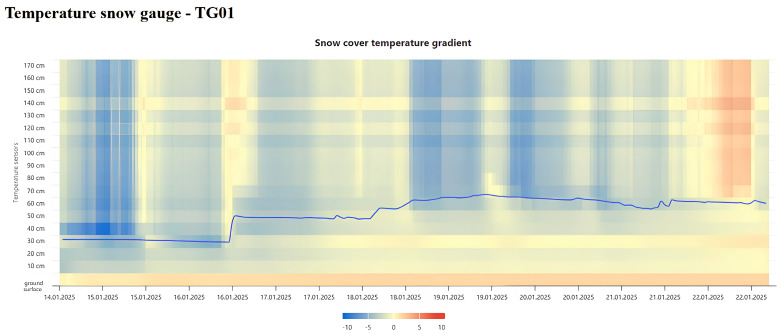
Example of snow cover temperature gradient of snow measuring rod.

For visual representation and data analysis, the study involved constructing a temperature gradient along the thickness of the snow cover. The figure shows sharp temperature fluctuations within the snowpack, which may influence the formation and triggering of spontaneous avalanches (Figure 7).

Data analysis and processing in the monitoring system are performed using logistic regression. Regression analysis is one of the main statistical tools that allows determining the relationship between a set of input factors and a dependent variable. When constructing a regression model, coefficients are determined for each input variable, indicating the degree of influence of each factor on the output variable.

When predicting spontaneous avalanches, it is necessary to determine the probability of an avalanche event at a specific avalanche collection site based on weather conditions, which in this case act as input variables. Logistic regression is the most appropriate tool for solving this problem.

In our case, the output variable is the recorded occurrence of spontaneous avalanches on a given date at a specific avalanche collection site. We used the Loginom Community 7.2 statistical software package to perform logistic regression calculations. Within this package, a scenario was created for computing the logistic regression model. The created scenario connects to the developed database, extracts data, and uses it to train the logistic regression model.

The avalanche hazard analysis and forecasting module, based on logistic regression and implemented using the Loginom Cimmunity 7.2 software platform, is integrated into the developed information system and operates using the previously described database and system hardware.

**Figure 8:**
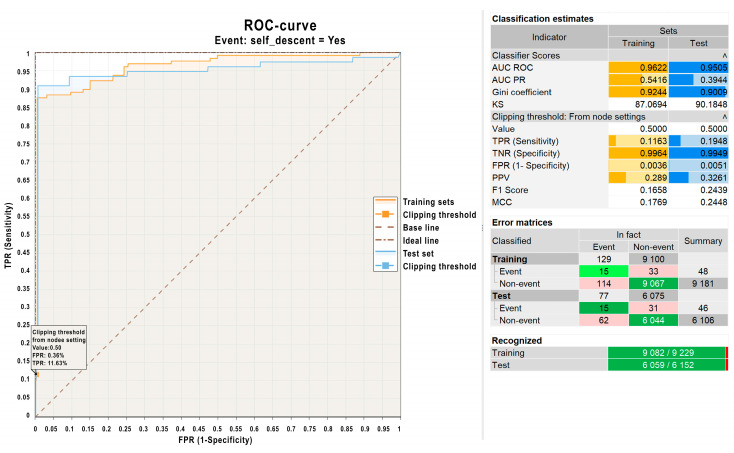
Quality assessment of the regression model.

Additionally, the statistical package was used to evaluate the quality of the constructed logistic regression model. Figure 8 shows a graph and corresponding data for the model evaluation. As can be seen from Figure 8, the constructed regression model for predicting avalanches demonstrates high quality, as evidenced by the following indicators: a high AUC-ROC value: 0.9622 for the training set and 0.9505 for the test set (values above 0.9 indicate excellent predictive performance); high recognition accuracy: over 98% for the training set (9082 out of 9229) and the test set (6059 out of 6152).

The logistic regression model developed in this part of the study can be used in the future to predict spontaneous avalanches based on meteorological observations in avalanche-prone areas of the East Kazakhstan region.

## 4. Conclusions

As a result of the project, a prototype avalanche hazard monitoring system was developed and tested, integrating a snow avalanche station and a temperature measuring rod. The system is a comprehensive solution based on advanced technologies and data collection methods. The snow avalanche station, equipped with modern sensors and the Ser[LOG] Plus recorder, provides reliable collection of weather data necessary for accurate prediction of avalanche activity. The temperature snow measuring rod, equipped with a laser rangefinder and CubeCell (Heltec Automation, Chengdu, China) microcontrollers, enables precise measurement of snow cover depth and the thermal stratification of the snowpack.

The use of solar energy and batteries to ensure the system’s autonomy makes it highly effective in remote and hard-to-reach areas. All collected data are transmitted to the AvaAPI service using LTE and Wi-Fi network technologies, ensuring prompt processing and availability of information for further analysis and decision making.

The developed avalanche hazard monitoring system has significant potential for further development and application both in Kazakhstan and internationally. Its modular structure and flexible architecture allow for the installation of additional sensors and adaptation to various terrain and climatic conditions. Furthermore, the integration of the monitoring system with machine learning algorithms for avalanche prediction can further enhance the accuracy and responsiveness of hazard forecasts.

The system’s ability to provide real-time data significantly contributes to improving the efficiency of avalanche warning dissemination, which is crucial for protecting human life and infrastructure in mountainous regions. Future work will focus on scaling the system to cover larger areas, integrating it with satellite remote sensing data, and improving predictive models by incorporating additional environmental parameters. Thus, the developed prototype represents an important step towards improving avalanche hazard monitoring systems, ensuring data accuracy, responsiveness, and opportunities for further expansion and integration with international initiatives in the field of climate monitoring and emergency prevention.

## Figures and Tables

**Table 1:** Comparative analysis of avalanche detection monitoring systems.

№	Country	Reference	Data	Methods/Models of Data Processing	Visualization	Automation
1	France	[1]	Three sets of data: an avalanche chronicle (Enquête Permanente sur les Avalanches, EPA), avalanche maps (Carte de Localization des Phénomènes d’Avalanche, CLPA) and a collection of data on hazards for human settlements.	Statistical methods	Geographic Information Systems (GIS)	no
2	China	[2]	Remote sensing and detailed field survey data.	Statistical methods combined with two machine learning models: logistic regression and multi-layered perceptron.	GIS	no
3	Northern Norway	[5]	Sentinel-1 satellite images for five years	Statistical methods for processing satellite images	GIS	no
4	Austria	[6]	Earth remote sensing data.	Processing video streams from webcams using a deep learning module	GIS	no
5	Austria	[7]	Distributed acoustic sensing data based on fiber-optic telecommunication cables	Statistical methods and machine learning methods	no	Automatic notification
6	Switzerland	[40]	The Silva Protect-CH project. Digital terrain models (DEM) with a resolution of 25 m	Numerical simulation of avalanches AVRIL-2D.	no	no
7	Switzerland	[37,38]	Data from automated snow and wind measuring stations (150 stations).	Statistical methods	-	Automatic notification
8	Italy	[8]	Data from automated measurement stations based on the use of infrasound waves.	A methodology for estimating threshold criteria for sources of infrasound waves and determining possible avalanches based on them.	-	Automatic notification
9	Türkiye	[10,11]	Digital terrain models (DEM) with a resolution of 10 m.	Numerical simulation of avalanches.	GIS	no
10	Russia	[35]	Data on slope parameters (slope topology) and meteorological indicators of snow cover stability in avalanche-prone locations.	Three-dimensional cellular model of snow accumulation (numerical modeling).	no	Automatic notification
11	Russia	[41]	AVYX^®^ radar data continuously scans slopes.	Statistical methods	no	Automatically sends a signal to any device
12	Russia	[42]	The Snow burst software and hardware complex. Meteorological data and snow cover temperature data.	Graph-analytical data processing platform and statistical data processing.	Grafana	Support and decision-making system

**Table 2:** Station equipment.

№	Name	Description	Selection Criteria or Important Characteristics
1	Ser[LOG] Plus	Ser[LOG]—Data Logger is a device designed to record, store and analyze data coming from various sensors, detectors or external sources. Power consumption: 10-30V DC. Interface: 5 × RS 485; 6 × RS 422; 4 × RS 232; USB-Device; USB-Host; Ethernet. Supported protocols: SDI-12, Modbus RTU, Modbus TCP, NMEAData sending protocols: HTTPS, MQTT.	The presence of analog inputs (for anemometers with a current loop), digital inputs, the ability to connect a communication module (the presence of an Ethernet port).
2	Wind sensor DBM-6410 (Magnetoelectric wind sensor)	The DBM-6410 wind sensor is used to measure air flow speed and wind direction. It is designed for autonomous operation or as part of meteorological stations and complexes. The DBM-6410 is indispensable for work in the field of climate measurements, meteorology, hydrology, ecology and other services. Protocols: Modbus-RTU, NMEA-0183.	The presence of an analog or digital output (RS485 + ARMY, CAR, NMEA protocol stack). Resistance to harsh weather conditions, resistant to icing.
3	Temperature-Humidity-Pressure Sensor THP[pro]	A multifunctional device designed to measure temperature, humidity and atmospheric pressure. This sensor is used in various applications, including environmental monitoring, climate control systems, smart buildings, weather stations and other areas where accurate measurement of these parameters is required. Protocols: SDI-12, NMEA, Modbus.	Operation using the SDI-12 protocol, the ability to configure for Modbus. Measurement of all 3 parameters at once, the presence of a digital output. Shelter, for protection from external influences.
4	Solar panel OSDA Solar 380M ODA380-30-MH (Half-Cell)	Monocrystalline solar panel. Quality class Grade A, Maximum power (P max)—380 W. Operating temperature range, °C—from −40 to +85	Matching the power consumption.
5	Battery VEKTOR GPL 12-75	Battery type—AGM, Battery capacity (C20), Ah—75, Nominal voltage-battery, V—12, Internal resistance, mOhm—6, Self-discharge, %—3, Terminal type—Under bolt Mb (Tb, T7, T13, B4, B5), Maximum charging current—22.5 A, Maximum discharge current—750 A, Discharge temperature range, °C—from −20 to +60	Stable operation at low temperatures. Matching the power consumption.
6	SRNE SR-ML2420 MPPT Solar Charge Controller	MPPT solar charge controller for 20 A, designed for 12/24 V photovoltaic systems. Maximum solar panel power is 260/520 W. The controller optimizes the charging process, preventing battery overcharging.	Stable operation at low temperatures.
7	Automatic switch VA 47-29	They are designed to protect distribution and group circuits with different loads.	Stable operation at low temperatures.
8	Time relay 12-240V AC/DC IEK	Designed for automatic switching on of electrical equipment with a delay after the switching command is given.	Stable operation at low temperatures.
9	LTE/Wi-Fi router iRZ RL25w	4G router with 2 SIM cards for automation systems with RS-232 and RS-485 interfaces, 4 LAN ports, 15 GPIO pins and built-in Wi-Fi. The multifunctional iRZ RL25w router is designed to transmit data over cellular networks.	Stable operation at low temperatures.
10	Outdoor all-weather cabinet 12U	Designed to accommodate autonomously functioning active and passive equipment, maintaining a specified temperature regime inside the cabinet during operation	Tightness, thermal insulation, preventing unauthorized access.
11	DC-DC converter	To ensure stable power supply for the Heltec LoRa 32 microcontroller integrated into the monitoring system, a DC-DC converter is used to reduce the voltage from 12 V supplied by the battery to 5 V.	Stable operation at low temperatures.
12	Antenna mast structure	A truss mast is a set of 3 m long aluminum sections with a triangular cross-section. Truss mast structures are used to use heavy-duty attachments, and can withstand extreme weather conditions such as strong winds, snow loads, and temperature fluctuations, which is extremely important in mountainous conditions.	Lightweight, prefabricated construction.

**Table 3:** The code for LTE/WiFi router.

import socket import ssl from struct import pack, unpack, unpack_from def send_via_ssl_socket(packet: bytes): HOST, PORT = “ApiHost”, 0001 sock = socket.socket(socket.AF_INET, socket.SOCK_STREAM) sock.settimeout(10) sock.connect((HOST, PORT)) sock.send(packet.encode(“utf-8”)) sock.close() ssock = socket.socket(socket.AF_INET, socket.SOCK_STREAM) ssock.bind((“0.0.0.0”, 5000)) ssock.listen() try: while True:	con, addr = ssock.accept() packet = con.recv(1024).decode().replace(“Host: 192.168.1.11:5000”, “Host: ApiHost:0001”) con.send( “““HTTP/1.1 200 OK Server: Werkzeug/3.0.1 Python/3.10.12 Date: Wed, 04 Dec 2024 07:00:46 GMT Content-Type: text/html; charset=utf-8 Content-Length: 4 Connection: close true””” .encode(“utf-8”) ) send_via_ssl_socket(packet) except KeyboardInterrupt: ssock.close()

**Table 4:** The software code of the microcontroller.

#include “LoRaWan_APP.h” #include “Arduino.h” #include “softSerial.h” #include <OneWire.h> #include <DallasTemperature.h> #include <Regexp.h>— #define ONE_WIRE_BUS GPIO4 OneWire oneWire(ONE_WIRE_BUS); DallasTemperature sensors(&oneWire); int deviceCount = 0; float tempC; char txpacket[BUFFER_SIZE]; char rxpacket[BUFFER_SIZE]; static RadioEvents_t RadioEvents; float txNumber; bool lora_idle=true; const String UID = “t1”; softSerial ss(GPIO5, GPIO0); String query_laser() { ss.begin(19200); String distance; while(true) { ss.write(‘D’); distance = ss.readString(); MatchState ms; char c_dist[50]; distance.toCharArray(c_dist, sizeof(c_dist)); ms.Target(c_dist); if(ms.Match(“(([0-9]\.[0-9]+\m\,[0-9]+))”) > 0) { Serial.println(“Got normal reading”); Serial.println(distance); distance = distance.substring(distance.indexOf(‘ ’), distance.length() - 1); break; } } ss.end();	return distance; } String ds18b20_query(){ String result = ““; sensors.begin(); deviceCount = sensors.getDeviceCount(); if (deviceCount > 0){ sensors.requestTemperatures(); for (int i = 0; i < deviceCount; i++) { tempC = sensors.getTempCByIndex(i); result += (String)(i+1) + “:” + (String)tempC + “;”; } } return result; } void loop() { if(lora_idle == true) { digitalWrite(Vext, LOW); String temperatures = ds18b20_query(); Serial.println(temperatures); txNumber += 0.01; String distance = query_laser(); distance.trim(); String voltage = (String)((float)getBatteryVoltage() / 1000.0); turnOnRGB(COLOR_SEND,0); Radio.Send( (uint8_t *)txpacket, strlen(txpacket) ); lora_idle = false; delay(900000); } } void OnTxDone( void ) { turnOffRGB(); lora_idle = true; } void OnTxTimeout( void ) { turnOffRGB(); Radio.Sleep( ); lora_idle = true; }

**Table 5:** The code for data receiver.

#define HELTEC_POWER_BUTTON #include <heltec_unofficial.h> #include <WiFi.h> #include <HTTPClient.h> #include <SoftwareSerial.h> #include <base64.h> SoftwareSerial ss(41,); String identifier = “id”; // Change this for each new base station const char* ssid = “net”; const char* password = “pass”; String target_url = “url”; String api_key = “apikey”; volatile bool rxFlag = false; long counter = 0; uint64_t last_tx = 0; uint64_t tx_time; uint64_t minimum_pause; String base64_output; String query_laser() { ss.begin(19200); ss.write(‘D’); String distance = ss.readString(); ss.end(); return distance; } void send_post_request(String packet) { if (WiFi.status() != WL_CONNECTED) { WiFi.disconnect(); WiFi.reconnect(); } if(WiFi.status() == WL_CONNECTED) { WiFiClient client; HTTPClient http; http.begin(target_url);	http.addHeader(“Content-Type”, “application/json”); http.addHeader(“Api-Key”, api_key); int httpResponseCode = http.POST(“{\”packet\”:\”“ + (String)packet + “\”}”); http.end(); } } void loop() { heltec_loop(); String rxdata; if (rxFlag) { rxFlag = false; radio.readData(rxdata); if (_radiolib_status == RADIOLIB_ERR_NONE) { laser_data.trim(); String packet = (String)”BEG “ + (String)”IB: “ + (String)identifier + “;” + (String)rxdata + “BD:” + laser_data + “ END” ; base64_output = base64::encode(packet); send_post_request(base64_output); } RADIOLIB_OR_HALT(radio.startReceive (RADIOLIB_SX126X_RX_TIMEOUT_INF)); } } void rx() { rxFlag = true; }

## Data Availability

Data are contained within the article. The data presented in this study are available from the corresponding author upon reasonable request.
